# Home-Based Psychiatric Outpatient Care Through Videoconferencing for Depression: A Randomized Controlled Follow-Up Trial

**DOI:** 10.2196/mental.5675

**Published:** 2016-08-03

**Authors:** Ines Hungerbuehler, Leandro Valiengo, Alexandre A Loch, Wulf Rössler, Wagner F Gattaz

**Affiliations:** ^1^ Laboratory of Neuroscience Institute of Psychiatry University of Sao Paulo São Paulo Brazil; ^2^ Department of Psychiatry Psychotherapy and Psychosomatics University Hospital of Psychiatry Zurich Zurich Switzerland

**Keywords:** telemedicine, telehealth, eHealth, videoconferencing, psychiatry, outpatient, home-care services, mental health, depression

## Abstract

**Background:**

There is a tremendous opportunity for innovative mental health care solutions such as psychiatric care through videoconferencing to increase the number of people who have access to quality care. However, studies are needed to generate empirical evidence on the use of psychiatric outpatient care via videoconferencing, particularly in low- and middle-income countries and clinically unsupervised settings.

**Objective:**

The objective of this study was to evaluate the effectiveness and feasibility of home-based treatment for mild depression through psychiatric consultations via videoconferencing.

**Methods:**

A randomized controlled trial with a 6- and 12-month follow-up including adults with mild depression treated in an ambulatory setting was conducted. In total, 107 participants were randomly allocated to the videoconferencing intervention group (n=53) or the face-to-face group (F2F; n=54). The groups did not differ with respect to demographic characteristics at baseline. The F2F group completed monthly follow-up consultations in person. The videoconferencing group received monthly follow-up consultations with a psychiatrist through videoconferencing at home. At baseline and after 6 and 12 months, in-person assessments were conducted with all participants. Clinical outcomes (severity of depression, mental health status, medication course, and relapses), satisfaction with treatment, therapeutic relationship, treatment adherence (appointment compliance and dropouts), and medication adherence were assessed.

**Results:**

The severity of depression decreased significantly over the 12-month follow-up in both the groups. There was a significant difference between groups regarding treatment outcomes throughout the follow-up period, with better results in the videoconferencing group. There were 4 relapses in the F2F group and only 1 in the videoconferencing group. No significant differences between groups regarding mental health status, satisfaction with treatment, therapeutic relationship, treatment adherence, or medication compliance were found. However, after 6 months, the rate of dropouts was significantly higher in the F2F group (18.5% vs 5.7% in the videoconferencing group, P<.05).

**Conclusions:**

Psychiatric treatment through videoconferencing in clinically unsupervised settings can be considered feasible and as effective as standard care (in-person treatment) for depressed outpatients with respect to clinical outcomes, patient satisfaction, therapeutic relationship, treatment adherence, and medication compliance. These results indicate the potential of telepsychiatry to extend access to psychiatric care to remote and underserved populations.

**ClinicalTrial:**

Clinicaltrials.gov NCT01901315; https://clinicaltrials.gov/ct2/show/NCT01901315 (Archived by WebCite at http://www.webcitation.org/6jBTrIVwg)

## Introduction

Depression affects approximately 350 million people worldwide and is the leading cause of disability, a major cause of morbidity, and thus a significant contributor to the global burden of disease at the social, economic, and clinical levels [1].

Antidepressant drugs and brief psychotherapy are effective, feasible and very cost-effective treatments for depression in primary health care settings [2]. However, less than half of those affected receive the care and support they need due to limited access to existing mental health care services [1]. The most common barriers include a lack of resources, the centralization of services in and near large cities and large institutions, low numbers of trained health care providers, inaccurate assessments, and social stigma [3]. The treatment gap is consistently worse in Low and Middle Income Countries (LMIC), where sometimes less than 10% of people in need receive treatment [1].

Information and communication technologies, such as mobile phones, apps and desktop software, and the Internet are able to overcome these barriers and reach a wide geographic area via remote delivery of care, thus expanding the reach of high-quality mental health care to patients who are otherwise unable to access it because of geographic location, transportation costs, or incapacitation due to serious physical or mental illness [4].

As verbal information and visual cues are the major and primary components of psychiatric treatment, the use of information and communication technologies such as live interactive videoconferencing is particularly well suited to psychiatric care. Unsurprisingly, the use of this technology in the field of psychiatry is over half a century old. In 1955, the first consultations via videoconferencing were conducted using a closed-circuit television system to transmit live therapy and education sessions via a macrowave link [5]. Due to technological advances in recent years and the tremendous global increase in Internet access and use of communication devices, including in remote and rural regions in LMIC—for example, with 108 million Internet users (53% of the population) in 2014, Brazil ranked fifth globally in the number of Internet users (behind China, the United States, India, and Japan) [6]—the provision of psychiatric treatment via videoconferencing, frequently called telepsychiatry, has become a viable method of delivering mental health care. Thus, telemental health services have been implemented around the world and are considered effective for the diagnosis and assessment of disorders in many populations (adult, child, geriatric, and different ethnicities) in many settings (emergency, home health), are comparable to in-person care, and complement other services in primary care [7].

According to the American Psychiatric Association, telepsychiatry is currently one of the most promising ways to increase access to psychiatric care for individuals living in underserved areas [8]. However, the number of randomized clinical trials is limited, and further studies are needed to generate empirical evidence for the large-scale use of psychiatric outpatient care via videoconferencing in high-, middle-, and low-income countries in clinically supervised and unsupervised (home-based) settings.

This study aims to verify the effectiveness and feasibility of a home-based treatment for mild depression through psychiatric consultations via videoconferencing.

## Methods

### Study Design and Setting

The study was designed as a parallel group, randomized controlled follow-up trial. With an allocation ratio of 1:1, outpatients were individually randomized to 2 different treatment conditions. Under the treatment as usual condition, participants underwent monthly face-to-face (F2F) consultations with their psychiatrists at the Institute of Psychiatry of the University of São Paulo Medical School. In the intervention condition, participants performed monthly home-based consultations with their psychiatrists using live interactive videoconferencing.

### Participants

To provide 80% power (5% level of significance) and assuming a medium effect size of 0.25 and a loss to follow-up of 25% after 12 months, the target sample size was 104 participants [9].

Participants were recruited at the Institute of Psychiatry (IPq) of the University of São Paulo Medical School and through public and social media announcements between May 2012 and April 2014. Interested individuals were prescreened by email to verify their place of residence, age, Internet access, and preexisting diagnoses or past psychiatric history. The Patient Health Questionnaire (PHQ-9) was used to screen for depression and to assess the severity of depression [10].

Individuals who lived in São Paulo and the surrounding areas, were between the ages of 18 and 55 years, had broadband Internet access at home, and showed symptoms of depression (PHQ-9 ≥ 5) were assessed for eligibility. On the basis of at least 2 in-person screening consultations, a diagnosis of depression was established with the Mini International Neuropsychiatric Interview [11], medication treatment was initiated, and the degree of depression was established using the Hamilton Depression Rating Scale (HDRS) (Table 1) [12]. Those with a total score less than 17 on the HDRS were considered mildly depressed and thus able to be randomized [13]. Individuals were not randomized if they: (1) did not meet the aforementioned inclusion criteria, (2) missed the second screening consultation, (3) showed an increased risk of suicide, or (4) refused treatment (Table 1).

**Figure 1:**
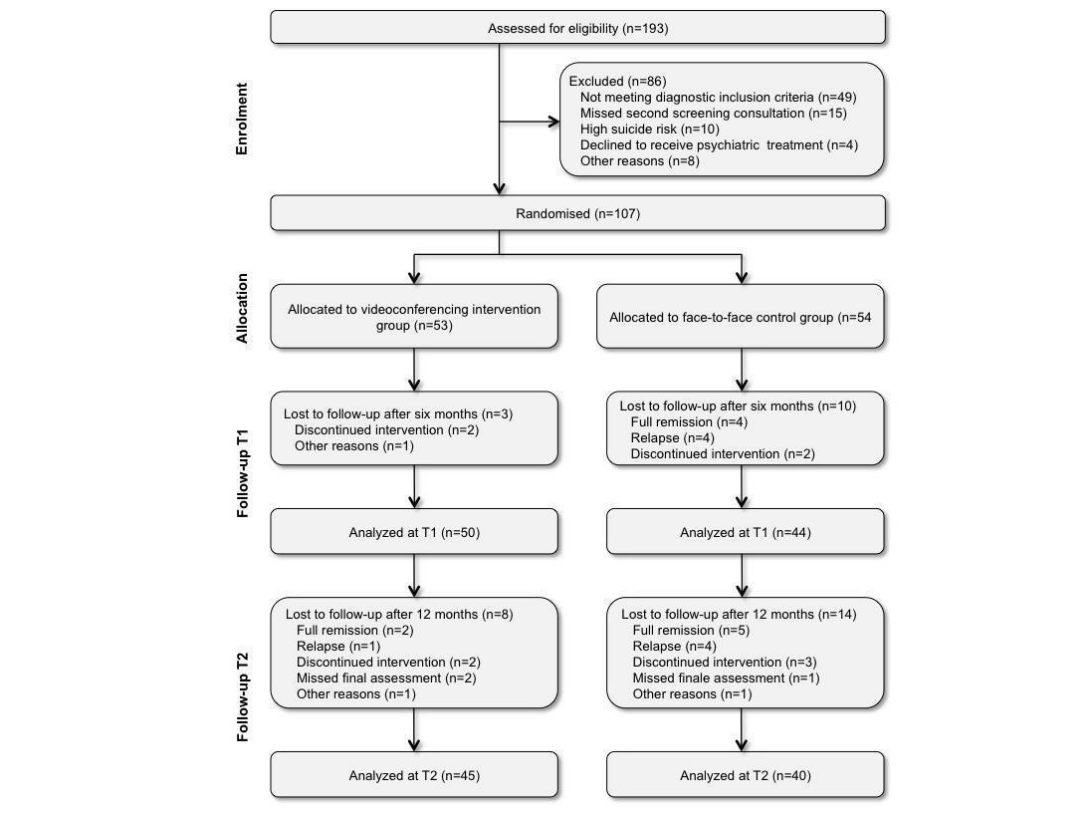
Sample flow diagram.

The allocation of participants to the treatment conditions (1:1) was conducted following a previously prepared randomization list in Excel. Fifty-three patients were randomized to the videoconferencing group and 54 to the F2F group. After 6 months, 3 patients in the videoconferencing group and 10 patients in the F2F group discontinued treatment. A total of 22 patients (8 in the videoconferencing group and 14 in the F2F group) were lost at second follow-up (Figure 1).

Discontinuation of treatment occurred if participants were fully remitted, missed 3 consultations in a row, had a relapse (HDRS > 17), needed additional care, or showed an elevated suicide risk. Even if they were excluded from the study, they continued receiving psychiatric treatment at the IPq.

### Intervention

All participants in both the groups concluded an in-person consultation at the beginning of the study (baseline), after 6 months (first follow-up) and after 12 months (second follow-up). In between those consultations, the control group received monthly psychiatric consultations in person at the psychiatric hospital, whereas the participants in the videoconferencing group underwent 5 home-based video consultations (once a month). Whereas patients in the F2F group received their medication right after each in-person consultation at the IPq, the medications for the patients treated via videoconferencing were delivered to the patient’s home.

The consultations and medication treatment decisions were individualized and determined by 7 psychiatrists with an average of 13 years of professional experiences who were trained in implementing consultations via videoconferencing and willing to deliver treatment in both conditions. Thus, all involved psychiatrists treated patients under both conditions. The consultations took approximately 20 minutes and included psychoeducation, medication monitoring, and counseling. During the entire study period, the participants were cared for by the same psychiatrist who conducted their initial screening consultation. Videoconferencing consultations were performed using the software Skype (Microsoft, Redmond, WA).

### Data Collection and Outcome Measures

At baseline and after 6 and 12 months, an in-person follow-up consultation was conducted with all participants, during which the severity of depression was assessed by the psychiatrists using the HDRS-17. After the consultation, the participants completed an automatically generated Web-based questionnaire that measured mental health status, satisfaction with treatment, therapeutic relationship, and medication compliance. The Web-based questionnaire was provided by the Web-based survey platform SurveyMonkey [14]. The responses were sent over a secure, Secure Sockets Layer–encrypted connection. The account was HIPAA-compliant and protected with a password. Only the study coordinator was allowed to access and export the collected data; the psychiatrists were blind to the ratings.

The clinical outcomes of this study were the severity of depression and self-reported mental health status, measured by the Mental Health Inventory (MHI-38) [15] and the number of relapses and medication history.

Additional outcome measures included treatment adherence, validated by the number of missed appointments and dropouts, medication compliance, satisfaction with treatment, and working alliance, measured by the following self-reported instruments: Morisky Medication Adherence Scale (MMAS-4) [16], Client Satisfaction Questionnaire (CSQ-8) [17], and the short version of the Working Alliance Inventory (WAI-S) [18].

### Statistical Analyses

Descriptive results—mean (SD)—of the demographic characteristics and the total outcomes scores at baseline and first and second follow-up (with 95% CI) are presented for each treatment group and were compared between groups (treatment effect) using *t*-tests, Wilcoxon–Mann–Whitney tests or Fisher’s exact tests.

To analyze changes over time in each group (time effect), one-way analysis of variance (ANOVA) was calculated for each outcome measure. To compare changes over time in the outcome measures between the F2F and videoconferencing group (treatment × time effect), repeated-measures ANOVA with a group–time interaction was performed. All statistical analyses were conducted using SPSS software, version 21.0 (IBM Corp.).

### Ethical Considerations

The trial protocol was approved by the local ethics committee and registered at clinicalTrials.gov (identifier NCT01901315). The study was conducted at a single location at the Institute of Psychiatry of the University of São Paulo Medical School. Written consent was obtained from all participants before randomization.

## Results

### Baseline Participant Characteristics

The treatment groups did not significantly differ in demographic characteristics (Table 1). Most of the participants were taking antidepressants (98%) within the recommended dosages, sometimes combined with anxiolytics and sedatives (40%). The most prescribed antidepressant was sertraline (34%-45%), followed by fluoxetine (18%-27%) and venlafaxine (14%-24%). No group differences were found regarding the type and dosage of medication at baseline.

On average, participants spent 3 hours traveling from their residence to the psychiatric hospital and back. This did not include waiting time at the hospital and the consultation itself. The groups were balanced regarding time spent on traveling. To attend a consultation via videoconferencing, the participants spent, on average, 30 minutes.

The groups were not balanced with regard to the severity of depression (mean F2F group score: 6.19 (3.61); mean videoconferencing group score: 7.92 (3.59); *P*=.01) or mental health status (mean F2F group score: 132.89 (25.49); mean videoconferencing group score: 121.25 (26.19); *P*=.02) at baseline. That is to say, the videoconferencing group showed significantly higher levels of depression and lower levels regarding mental health status than the F2F group at baseline.

**Table 1:** Baseline characteristics of participants in each treatment arm.

Baseline Variables		Total (n=107)	VC^a^(n=53)	F2F^b^(n=54)
Age, mean (SD^c^), year		35.64 ± 8.33	35.42 ± 8.18	35.87 ± 8.53
Female (%)		76 (71.0%)	39 (73.6%)	37 (68.5%)
Brazilian (%)		103 (96.3%)	52 (98.1%)	51 (94.4%)
Marital Status (No., %)				
	Single	58 (54.2%)	25 (47.2%)	33 (61.1%)
	Married	34 (31.8%)	22 (41.5%)	12 (22.2%)
	Divorced	13 (12.1%)	5 (9.4%)	8 (14.9%)
	Widowed	2 (1.9%)	1 (1.9%)	1 (1.9%)
Education (No., %)				
	Primary	2 (1.9%)	0 (0.0%)	2 (3.7%)
	Secondary	30 (28.0%)	14 (26.4%)	16 (29.6%)
	Higher	75 (70.1%)	39 (73.6%)	36 (66.7%)
Working situation (No., %)				
	Student/Homemaker	13 (12.1%)	6 (11.3%)	3 (5.7%)
	Employed	67 (62.6%)	32 (60.4%)	35 (66.0%)
	Unemployed	24 (22.4%)	11 (20.8%)	12 (22.6%)
	Retired	1 (0.9%)	0 (0.0%)	1 (1.9%)
	Other	10 (9.3%)	4 (7.5%)	2 (3.8%)
Severity of depression, mean (SD)		7.05 ± 3.69	7.92 ± 3.59	6.19 ± 3.61
Mental health status, mean (SD)		127.12 ± 26.37	121.25 ± 26.19	132.89 ± 25.49
Medication – multiple choices; No. (%)				
	Antidepressants	105 (98.1%)	52 (98.1%)	53 (98.1%)
	Tranquilizers	29 (27.1%)	13 (24.5%)	16 (26.6%)
	Mood stabilizers	2 (1.8%)	1 (1.9%)	1 (1.9%)
	Antipsychotics	1 (0.9%)	1 (1.9%)	0 (0%)
	Other	7 (6.5%)	2 (3.8%)	5 (9.4%)

^a^ VC: Videoconferencing.

^b^ F2F: Face-to-face.

^c^ SD: standard deviation

### Outcome Measures

Within the 35-month period of study, a total of 950 consultations were delivered (489 via videoconferencing and 461 F2F), and 286 assessments were conducted. The follow-up data are summarized in Table 2.

There were no differences in demographic variables, severity of depression, or mental health status at baseline between those who dropped out and those who did not.

**Table 2:** Unadjusted mean scores and standard deviations of outcome measures.

Measure by Time Point		Score					
		VC^a^		F2F^b^		Total	
		Mean (SD)	No.	Mean (SD)	No.	Mean (SD)	No.
HDRS^c^							
	Baseline	7.92 (3.59)	53	6.19 (3.60)	54	7.05 (3.69)	107
	Follow-up: 6 months	4.65 (4.13)	49	3.90 (3.88)	42	4.31 (4.01)	91
	Follow-up: 12 months	3.42 (3.58)	45	4.45 (4.13)	40	3.90 (3.86)	85
MHI^d^							
	Baseline	121.25 (26.19)	53	132.89 (25.49)	54	127.12 (26.37)	107
	Follow-up: 6 months	123.98 (27.56)	50	117.09 (25.54)	44	120.76 (26.72)	94
	Follow-up: 12 months	137.71 (28.88)	45	143.32 (25.07)	40	140.35 (27.14)	85
CSQ^e^							
	Baseline	27.66 (2.82)	53	28.43(2.61)	54	28.05 (2.73)	107
	Follow-up: 6 months	28.24 (3.06)	50	29.45 (2.21)	44	28.81 (2.75)	94
	Follow-up: 12 months	27.91 (3.76)	45	29.35 (2.48)	40	28.59 (3.28)	85
WAI^f^							
	Baseline	68.90 (12.56)	52	72.11 (10.26)	54	70.54 (11.50)	106
	Follow-up: 6 months	63.52 (8.86)	50	64.93 (8.95)	44	64.18 (8.88)	94
	Follow-up: 12 months	70.78 (11.96)	45	73.41 (9.18)	39	72.00 (10.78)	84

^a^ VC: videoconferencing.

^b^ F2F: face-to-face.

^c^ HDRS: Hamilton Depression Rating Scale.

^d^ MHI: Mental Health Inventory.

^e^ CSQ: Client Satisfaction Questionnaire.

^f^**WAI: Working** Alliance Inventory.

### Clinical Outcomes

At 6 and 12 months, the initial group differences with respect to severity of depression and mental health status were no longer significant. Each group showed a significant decrease in the severity of depression (videoconferencing: F_2_ = 26.57, *P*<.001; F2F: F_2_ = 29.99, *P*<.001) and a significant increase in mental health status (videoconferencing: F_1.426_ = 4.86, *P*=.02; F2F: F_1.437_ = 9.17, *P*=.001) over the study period. The repeated-measures ANOVA showed a statistically significant interaction between treatment and time regarding the severity of depression (F_2_ = 6.12, *P*=.003). The estimated partial-η2 for the interaction of time and treatment was 0.24.

Most of the participants continued taking antidepressants at 6 (95%) and 12 months (79%) within the recommended dosages, combined with sedatives (40%). No group differences were found with respect to the type and dosage of medication at the 6- and 12-month follow-up.

Five participants were excluded because they relapsed (scored higher than 17 on the HDRS); 4 in the F2F and 1 in the videoconferencing group.

### Treatment Adherence and Medication Compliance

The dropouts did not differ significantly from the completers in demographic variables, degree of depression, or mental health status at baseline.

At 6 months, there were significantly more dropouts in the F2F group (n=10) than in the videoconferencing group (n=3; X^2^_1_ = 4.143, *P*=.04). At 12 months, there were still more dropouts in the F2F group (n=14) than in the videoconferencing group (n=8), but the difference was no longer significant.

Moreover, participants in the F2F group also tended to miss more appointments than participants in the videoconferencing group (F_105_ = 0.753, *P*=.06).

On average, 30% of the participants were adherent to their medication. There were no significant group differences regarding medication compliance at 6 and 12 months between the 2 groups. Participants in the F2F group tended to be more adherent than participants in the videoconferencing group at 12 months (X^2^_1_ = 2.864, *P*=.07).

### Satisfaction With Treatment

There were no significant differences between treatment conditions with respect to satisfaction of the participant at 6 and 12 months. Overall, satisfaction significantly increased during the first 6 months among the whole sample (Z=−2.031, *P*=.04) and remained stable until the end of the study. There were no significant changes in satisfaction over the entire study period, and the repeated-measures ANOVA did not show a significant interaction between treatment condition and time.

### Working Alliance

Similar to satisfaction, there were no group differences with respect to working alliance at either of the follow-ups. Both the groups showed a significant increase in working alliance during the 12 months of treatment (videoconferencing: F_2_ = 11.11, *P*<.001; F2F: F_2_ = 29.23, *P*<.001). There were no significant differences between groups regarding changes in the Working Alliance Inventory scores (repeated-measures ANOVA).

## Discussion

### Principal Findings

This study constitutes the first randomized clinical trial to evaluate the effectiveness and feasibility of home-based general psychiatric outpatient care via videoconferencing.

The implementation of the study was rigorously controlled, including sample size calculations, the use of standardized assessment instruments, monitoring of treatment and medication delivery, follow-up assessments up to 12 months, and a high participant adherence (79% completed 1 year of treatment).

The results are mostly in line with previous studies in clinically supervised settings, which compared F2F with videoconferencing treatment among patients with different psychiatric diagnoses. Most of those studies also did not find any significant differences regarding clinical outcomes between the 2 treatment conditions, measured by symptom severity, medication history, duration of inpatient treatment, mental health status, global functioning, or neuropsychological outcomes [19-27]. Self-reported satisfaction with treatment [21,23,27-29] and treatment adherence—assessed in terms of compliance, dropout rates, number of appointments kept, and pill counts [23,25]—were also comparable between F2F and videoconferencing psychiatric treatments.

Moreover, patients in the videoconferencing group were able to establish an equivalent therapeutic relationship as those treated in person in this study. This was also shown in a study with male inmates suffering from different psychiatric disorders [21].

However, in this study, the improvement in severity of depression was even greater among participants treated via videoconferencing. Two previous studies including depressed low-income Hispanic participants also found that psychiatric consultations via videoconferencing generated better clinical outcomes than usual (in person) care [25,30]. However, it has to be considered that in this study, the videoconferencing group started with a significantly higher score on the HDRS and thus had a greater potential to decrease. Moreover, the differences between groups, even at baseline, were only between 1 and 2 points; however, given the low HDRS total scores at 6 and 12 months (< 5), this difference has limited clinical relevance.

Another noteworthy result was the significantly lower dropout rate in the videoconferencing group after 6 months. Most of the participants travelled up to 3 hours to attend an in-person consultation at the psychiatric hospital. This typical long travel time is due to traffic and the limited public transportation system in São Paulo, and thus, the considerable time saved among patients treated by videoconferencing could be an explanation of the higher dropout rate in the F2F group.

### Limitations

A major strength of this study was that it was conducted in a setting in which telepsychiatry is most likely to be used. However, the individualized treatment and thus the naturalistic nature of the service also produced limitations regarding the replicability and comparability of the results.

Another strength and, at the same time, limitation of the study was that all psychiatrists delivered F2F and videoconferencing consultations. The psychiatrists were instructed to provide the same type and level of service to patients seen in person and through videoconferencing. Nevertheless, a bias in terms of psychiatrist’s favoring one method over another could have influenced the findings.

Moreover, there are advantages and disadvantages to using Skype in clinical settings. The advantages are its familiarity and ease of access; the disadvantages are security concerns. However, Skype uses a 256-bit encryption, which meets the Advanced Encryption Standard specified by the US National Institute of Standard Technology. Furthermore, no firm evidence either in favor of or against the use of Skype for clinical telehealth has been found thus far [31].

Most of the scales used in this study have been translated, adapted, and validated in the Brazilian population [32-35]. For the assessment of satisfaction, the official CSQ in (Brazilian) Portuguese was used [17], which has not been validated yet. Moreover, at the time the study was planned, no adequate scale measuring medication adherence was validated in a Brazilian Portuguese version. However, a translated version in Brazilian Portuguese of the MMAS-8 [16] scale was available, and thus, a short form was created based on this translation [36].

Another limitation of this study is that there was no intention-to-treat analysis. However, if a subject who actually did not receive the same or any treatment is included as a subject who received the whole treatment, then the results indicate very little about the efficacy of the treatment [37].

### Conclusion

Based on the findings, psychiatric consultations via videoconferencing can be considered applicable for the home-based treatment of mildly depressed patients and as effective as F2F treatment with respect to clinical outcomes, treatment adherence, medication compliance, satisfaction, and working alliance.

Despite being largely successful elsewhere, telepsychiatry has yet to make its mark in LMIC of the developing world [38]. This study demonstrates the successful implementation and evaluation of treatment delivery for this population in a resource-limited setting. Moreover, it shows that home-based mental health care has the potential to provide effective treatment to many individuals who may not otherwise seek help, due to geographic or economic barriers or perceived stigma of receiving mental health treatment.

Further randomized clinical studies are needed to provide empirical evidence on the feasibility and treatment efficacy over time of large-scale, sustainable psychiatric outpatient care. The integration of videoconferencing as a routine component of psychiatric care would benefit patients through increased access to needed treatment and would thus help reduce the treatment gap in LMIC and in many industrialized countries.

## Abbreviations

CSQ: Client Satisfaction Questionnaire
F2F: face-to-face
HDRS: Hamilton Depression Rating Scale
HIPAA: Health Insurance Portability and Accountability Act
IPq: Institute of Psychiatry
MHI: Mental Health Inventory
PHQ-9: Patient Health Questionnaire
SD: standard deviation

## References

[1] Marcus M, Yasamy MT, van Ommeren M, Chisholm D, Saxena S World Health Organization: Mental health.

[2] World Health Organization.

[3] Buckner TA, Scheffler RM, Shen G, Yoon J, Chisholm D, Morris J, Fulton BD, Dal Poz MR, Saxena S (2011). The mental health workforce gap in low- and middle-income countries: a needs-based approach. Bulletin of the World Health Organization.

[4] (2011). World Health Organization.

[5] Wittson CL, Benschoter R (1972). Two-way television: helping the Medical Center reach out. Am J Psychiatry.

[6] (2014). Internet Users by Country.

[7] Hilty DM, Ferrer DC, Parish MB, Johnston B, Callahan EJ, Yellowlees PM (2013). The effectiveness of telemental health: a 2013 review. Telemed J E Health.

[8] American Psychiatric Association.

[9] Faul F, Erdfelder E, Lang AG, Buchner A (2007). G*Power 3: a flexible statistical power analysis program for the social, behavioral, and biomedical sciences. Behav Res Methods.

[10] de Lima Osório F, Vilela Mendes A, Crippa JA, Loureiro SR (2009). Study of the discriminative validity of the PHQ-9 and PHQ-2 in a sample of Brazilian women in the context of primary health care. Perspect Psychiatr Care.

[11] Sheehan DV, Lecrubier Y, Sheehan KH, Amorim P, Janavs J, Weiller E, Hergueta T, Baker R, Dunbar GC (1998). The Mini-International Neuropsychiatric Interview (M.I.N.I.): the development and validation of a structured diagnostic psychiatric interview for DSM-IV and ICD-10. J Clin Psychiatry.

[12] Hamilton M (1960). A rating scale for depression. J Neurol Neurosurg Psychiatry.

[13] Moreno R, Moreno D (1998). Escalas de depressão de Montgomery & Åsberg (MADRS) e de Hamilton (HAM–D). Rev Psiq Clin.

[14] SurveyMonkey Inc.

[15] Brook RH, Ware JE Jr, Davies-Avery A, Stewart AL, Donald CA, Rogers WH, Williams KN, Johnston SA (1979). Overview of adult health measures fielded in Rand's health insurance study. Med Care.

[16] Morisky DE, Green LW, Levine DM (1986). Concurrent and predictive validity of a self-reported measure of medication adherence. Med Care.

[17] Attkisson CC, Zwick R (1982). The client satisfaction questionnaire. Psychometric properties and correlations with service utilization and psychotherapy outcome. Eval Program Plann.

[18] Tracey TJ, Kokotovic AM (1989). Factor structure of the Working Alliance Inventory. Psychological Assessment: A Journal of Consulting and Clinical Psychology.

[19] Modai I, Jabarin M, Kurs R, Barak P, Hanan I, Kitain L (2006). Cost effectiveness, safety, and satisfaction with video telepsychiatry versus face-to-face care in ambulatory settings. Telemed J E Health.

[20] Kennedy C, Yellowlees P (2003). The effectiveness of telepsychiatry measured using the Health of the Nation Outcome Scale and the Mental Health Inventory. J Telemed Telecare.

[21] Morgan RD, Patrick AR, Magaletta PR (2008). Does the use of telemental health alter the treatment experience? Inmates' perceptions of telemental health versus face-to-face treatment modalities. J Consult Clin Psychol.

[22] Grady BJ, Melcer T (2005). A retrospective evaluation of TeleMental Healthcare services for remote military populations. Telemed J E Health.

[23] Ruskin PE, Silver-Aylaian M, Kling MA, Reed SA, Bradham DD, Hebel JR, Barrett D, Knowles F 3rd, Hauser P (2004). Treatment outcomes in depression: comparison of remote treatment through telepsychiatry to in-person treatment. Am J Psychiatry.

[24] Poon P, Hui E, Dai D, Kwok T, Woo J (2005). Cognitive intervention for community-dwelling older persons with memory problems: telemedicine versus face-to-face treatment. Int J Geriatr Psychiatry.

[25] Chong J, Moreno F (2012). Feasibility and acceptability of clinic-based telepsychiatry for low-income Hispanic primary care patients. Telemed J E Health.

[26] De Las Cuevas C, Arredondo MT, Cabrera MF, Sulzenbacher H, Meise U (2006). Randomized clinical trial of telepsychiatry through videoconference versus face-to-face conventional psychiatric treatment. Telemed J E Health.

[27] O'Reilly R, Bishop J, Maddox K, Hutchinson L, Fisman M, Takhar J (2007). Is telepsychiatry equivalent to face-to-face psychiatry? Results from a randomized controlled equivalence trial. Psychiatr Serv.

[28] Elford R, White H, Bowering R, Ghandi A, Maddiggan B, St John K, House M, Harnett J, West R, Battcock A (2000). A randomized, controlled trial of child psychiatric assessments conducted using videoconferencing. J Telemed Telecare.

[29] Bishop JE, O'Reilly RL, Maddox K, Hutchinson LJ (2002). Client satisfaction in a feasibility study comparing face-to-face interviews with telepsychiatry. J Telemed Telecare.

[30] Moreno FA, Chong J, Dumbauld J, Humke M, Byreddy S (2012). Use of standard Webcam and Internet equipment for telepsychiatry treatment of depression among underserved Hispanics. Psychiatr Serv.

[31] Armfield NR, Gray LC, Smith AC (2012). Clinical use of Skype: a review of the evidence base. J Telemed Telecare.

[32] Freire MA, de Figueiredo VLM, Gomide A, Jansen K, da Silva RA, Magalhães P, Kapczinski FP (2014). Escala Hamilton: estudo das características psicométricas emu ma amostra do sul do Brasil. J Bras Psiquiatr.

[33] Ribeiro J (2001). Mental Health Inventory: Um estudo de adaptação à população portuguesa. Psicologia, Saúde & Doenças.

[34] Prado OZ, Meyer SB (2006). Avaliação da relação terapêutica na terapia assíncrona via internet. Psicol estud.

[35] Amorim P (2000). Mini International Neuropsychiatric Interview (MINI): validação de entrevista breve para diagnóstico de transtornos mentais. Rev Bras Psiquiatr.

[36] Oliveira-Filho AD, Barreto-Filho JA, Neves SJF, Lyra Junior DP (2012). Relação entre a Escala de Adesão Terapêutica de oito itens de Morisky (MMAS-8) e o controle da pressão arterial. Arq Bras Cardiol.

[37] Gupta SK (2011). Intention-to-treat concept: A review. Perspect Clin Res.

[38] Chakrabarti S (2015). Usefulness of telepsychiatry: A critical evaluation of videoconferencing-based approaches. World J Psychiatry.

